# Multigene testing panels reveal pathogenic variants in sporadic breast cancer patients in northern China

**DOI:** 10.3389/fgene.2023.1271710

**Published:** 2023-11-09

**Authors:** Yinfeng Liu, Jie Zheng, Yue Xu, Ji Lv, Zizheng Wu, Kai Feng, Jiani Liu, Weitao Yan, Liguang Wei, Jiangman Zhao, Lisha Jiang, Meng Han

**Affiliations:** ^1^ Breast Disease Diagnosis and Treatment Center, The First Hospital of Qinhuangdao, Qinhuangdao, Hebei, China; ^2^ Shanghai Biotecan Pharmaceuticals Co., Ltd., Shanghai, China

**Keywords:** breast cancer, multi-gene panels, rare genetic variants, *in silico* protein modeling, pathogenic/likely pathogenic variants

## Abstract

**Background:** Breast cancer, the most prevalent malignancy in women worldwide, presents diverse onset patterns and genetic backgrounds. This study aims to examine the genetic landscape and clinical implications of rare mutations in Chinese breast cancer patients.

**Methods:** Clinical data from 253 patients, including sporadic and familial cases, were analyzed. Comprehensive genomic profiling was performed, categorizing identified rare variants according to the American College of Medical Genetics (ACMG) guidelines. In silico protein modeling was used to analyze potentially pathogenic variants’ impact on protein structure and function.

**Results:** We detected 421 rare variants across patients. The most frequently mutated genes were *ALK* (22.2%), *BARD1* (15.6%), and *BRCA2* (15.0%). ACMG classification identified 7% of patients harboring Pathogenic/Likely Pathogenic (P/LP) variants, with one case displaying a pathogenic BRCA1 mutation linked to triple-negative breast cancer (TNBC). Also identified were two pathogenic *MUTYH* variants, previously associated with colon cancer but increasingly implicated in breast cancer. Variants of uncertain significance (VUS) were identified in 112 patients, with PTEN c.C804A showing the highest frequency. The role of these variants in sporadic breast cancer oncogenesis was suggested. In-depth exploration of previously unreported variants led to the identification of three potential pathogenic variants: *ATM c*.*C8573T*, *MSH3 c*.*A2723T*, *and CDKN1C c*.*C221T*. Their predicted impact on protein structure and stability suggests a functional role in cancer development.

**Conclusion:** This study reveals a comprehensive overview of the genetic variants landscape in Chinese breast cancer patients, highlighting the prevalence and potential implications of rare variants. We emphasize the value of comprehensive genomic profiling in breast cancer management and the necessity of continuous research into understanding the functional impacts of these variants.

## Introduction

Breast cancer (BC) is one of the most prevalent and deadly malignancies in women worldwide. Globally, BC is the most commonly diagnosed cancer among women, with an estimated 2.3 million new cases and nearly 685,000 deaths reported in 2020 (Arnold et al., 2022; Siegel et al., 2023). In China, the incidence rate of BC has been significantly rising, making it an alarming public health concern. It is currently the most common cancer among women in China, with about 367,900 new cases and 97,972 deaths reported in 2020 (Lei et al., 2021). This increasing trend necessitates further research into understanding the risk factors and genetic predispositions underlying BC in the Chinese population.

While environmental and lifestyle factors play a significant role in BC development, a growing body of evidence indicates that genetic susceptibility also contributes to the onset of this disease. Approximately 5%–10% of BC cases are hereditary, often attributed to inherited pathogenic or likely pathogenic (P/LP) variants in highly penetrant predisposition genes (Yoshimura et al., 2022). The *BRCA1* and *BRCA2* genes are the most well-known and extensively studied of these genes (Yoshimura et al., 2022). Mutations in these genes significantly increase the risk of breast and ovarian cancer (Zhong et al., 2015), and are also related to the prognosis and recurrence of breast cancer (Shah et al., 2016). A study by Zhang et al. found that among 30,223 adult participants of the BioMe Biobank, 218 (0.7%) individuals harbored expected PVs in *BRCA1/2* (Abul-Husn et al., 2019). However, mutations in these genes account for only a fraction of hereditary BC cases, suggesting the involvement of other genetic variants. In addition to *BRCA1* and *BRCA2*, several other susceptibility genes have been associated with BC, including *TP53*, *PALB2*, *CHEK2*, *ATM* and so on (Foulkes, 2021), indicating that the utilization of multi-gene panel testing could potentially confer enhanced benefits to patients (Bono et al., 2021).

Currently the majority of the current understanding of genetic susceptibility to BC is based on studies conducted in Western populations. The genetic landscape of BC in China, a country with a complex population structure and unique lifestyle factors, remains relatively unexplored. Preliminary studies suggest that the prevalence of inherited P/LP variants in highly penetrant predisposition genes among Chinese BC patients may be similar to global estimates. The occurrence of harmful *BRCA1/2* mutations in the broad Chinese population has been documented to vary between 0.29% and 1.10% (with *BRCA1* variations between 0.02% and 0.34% and *BRCA2* variations from 0.11% to 0.27%) (Lei et al., 2022). In the Chinese population, research on susceptibility genes beyond *BRCA1* and *BRCA2* is still evolving. In a cohort of 7,657 unselected BC patients who tested negative for *BRCA1/2* germline mutations, a multigene panel revealed that 29 cases (0.38%) carried harmful *RAD51D* germline mutations (Chen et al., 2018). A study by Li et al. have suggested the presence of additional susceptibility genes, such as *EFEMP1*, contributing to BC risk among Chinese women (Li et al., 2016).

Genetic diversity among different population in China may result in different gene-disease associations, emphasizing the need for population-specific genetic studies. In this study, we aim to fill a significant gap in the existing literature by examining the spectrum of hereditary gene variants that may trigger sporadic BC in the northern Chinese population by testing three different gene panels in a total of 253 patients.

## Materials and methods

### Patients and samples

A total of 253 women with breast cancer were recruited in Department of Breast Surgery of First Hospital of Qinhuangdao, from September 2020 to July 2022. Clinical information of participants was obtained from Electronic Medical Record System and questionnaire. 2–4 mL peripheral blood from each participant was collected by EDTA anticoagulated tube.

### DNA extraction and quality control

Genomic DNA (gDNA) of peripheral blood was extracted by QIAamp DNA Blood Mini Kit (QIAGEN GmbH), then quantity and purity were evaluated by Qubit 3.0 and NanoDrop 2000 spectrophotometer (Thermo Fisher Scientific, Wilmington, United States). 300 ng gDNA per sample was mechanically fragmented using an E220 Focused-ultrasonicator (Covaris, LLC., Massachusetts, United States). The Agilent 2100 Bioanalyzer instrument with Agilent High Sensitivity DNA Kit (Agilent Technologies, Inc., CA, United States) were used for sizing and quantitation of fragmented DNA. The targeted size of fragmented DNA was from 150 to 200 bp.

### Library preparation and sequencing

10–100 ng fragmented DNA was used for library construction using the SureSelect XT Low Input Reagent Kits (Agilent Technologies, Inc., CA, United States), including end-repair, dA-tail the 3′end of the DNA fragments, ligating the paired-end adaptor, and pre-amplification. 500–2000ug DNA of the whole genomic libraries were captured using Agilent SureSelect XT custom panel probes and finally amplified. Three panels were used throughout this study (Supplementary Table S1), encompassing 21 genes (97 patients; 38.3%), 37 genes (66 patients; 26.1%) and 64 genes (90 patients, 35.6%). The use of three panels is due to the upgrade in the panel’s detection capability. Patients who were recruited after the upgrade would use the new panel. After quality control and quantification by Agilent 2100 Bioanalyzer and Qubit 3.0, the libraries were sequenced on Illumina Nextseq CN500 platform (Illumina Inc., CA, United States) in PE150 mode. All the sequencing data were upload to SRA database with an accession number PRJNA998571.

### Bioinformatics analysis

Clean data was obtained following filtering adapter, low quality reads and reads with proportion of N>10%. Reads were aligned to the reference human genome (UCSC hg19) (Meyer et al., 2013) using the Burrows-Wheeler Aligner v. 0.7.17 (Li and Durbin, 2009). Next, the Picard and Genome Analysis Toolkit (GATK v.3.7) (McKenna et al., 2010) method was adopted for duplicate removal, local realignment and Base Quality Score Recalibration, and generated the quality statistics, including mapped reads, mean mapping quality and mean coverage. Finally, the GATK HaplotypeCaller was used for SNV and InDel identification.

Variants were annotated using the ANNOVAR software tool (Wang et al., 2010). Annotations for mutation function (including frameshift insertion/deletion, non-frameshift insertion/deletion, synonymous SNV, nonsynonymous SNV, stopgain, stoploss), mutation location [such as exonic, intronic, splicing, upstream, downstream, 3′untranslated region (UTR), 5′UTR and so on], amino acid changes, 1000 Genomes Project data, the Exome Aggregation Consortium (ExAC) data, the NHLBI Exome Sequencing Project (ESP) data and dbSNP reference number were performed. Mutation databases including HGMD (http://www.hgmd.cf.ac.uk/), and ClinVar (http://www.ncbi.nlm.nih.gov/clinvar/) were also included in the analysis pipeline. In this study, in order to making the interpretation of the sequence variants much more efficient and conclusive, annotated variants were filtered about mutation location (exonic and splicing region were reserved), mutation function (synonymous SNV and UNKOWN were removed) and allele frequency (≤0.05 AF in 1000 g, ESP and ExAC database).

### Germline mutation classification

All mutations were classified according to the American College of Medical Genetics (ACMG) professional practice and guidelines [five‐tier mutation: P (Pathogenic); LP (Likely Pathogenic); Variants of uncertain significance (VUS); LB (Likely Benign); and B (Benign)] (Richards et al., 2015). Mutation classification was generated by genetic Counselor and verified by two curators.

### Statistical analysis

Descriptive statistics were used to summarize the demographic and clinical characteristics of the participants. Categorical variables were analyzed using Chi-square tests, while continuous variables were examined using Student's t-tests or Mann-Whitney U tests as appropriate. All tests were two-sided, and a *p*-value <0.05 was considered statistically significant. All statistical analyses were performed using the R statistical software (version 4.0.2).

## Results

### Patient characteristics

The clinical profiles of 253 patients are detailed in Table 1. Among these, 242 cases were diagnosed with sporadic breast cancer, while the remaining 11 patients presented with a familial history of the disease. The median age at diagnosis across all patients was 58 years, with an interquartile range (IQR) of 48–65 years. The patient cohort was stratified into two groups based on the age at the time of diagnosis: 25 cases were classified as early-onset (diagnosed before 40 years of age), while the remaining 228 were designated as late-onset cases. Notably, three patients were diagnosed with bilateral breast cancer.

**TABLE 1:** Clinical characteristics of 253 breast cancer.

Clinical characteristics	Number (*n* = 253)	P/LP	Negative	*p*-value
Age				0.865
Early-onset (≤40 years old)	25 (9.88%)	1	24	
Late-onset (>40 years old)	228 (90.12%)	16	212	
Mean ± SD	56.37 ± 12.14	55.88 ± 12.70	56.40 ± 12.13	
Family history of breast cancer				
Yes	11 (4.35%)	1	10	1
No	242 (95.65%)	16	226	
Stage				
0–2	165 (65.22%)	13	152	0.93
3–4	35 (13.83%)	2	33	
Unknown	53 (20.95%)	2	51	
Molecular Subtypes				
Luminal A	71 (28.06%)	4	67	0.95
Luminal B	53 (20.95%)	4	49	
HER2-enriched	58 (22.92%)	4	54	
Triple-negative or basal-like	39 (15.42%)	4	35	
Unknown	32 (12.65%)	1	31	
Location				0.49
Bilateral	3 (1.19%)	1	2	
Unilateral	250 (98.81%)	16	234	

### Frequency of breast cancer predisposition genes with rare variants

**FIGURE 1:**
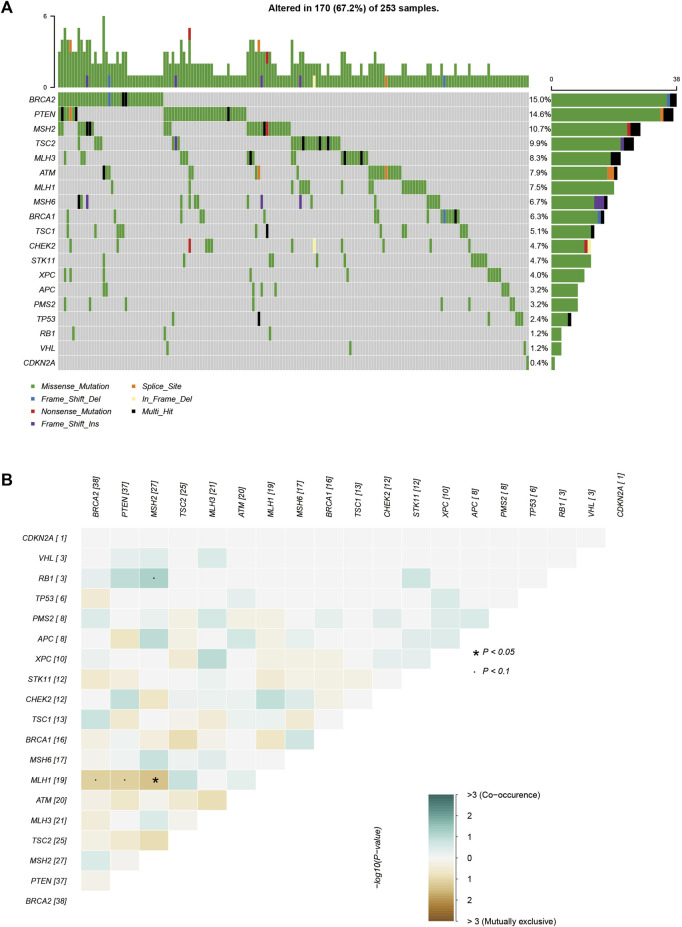
The mutations tested by 21-gene-panel in 253 cases. **(A)** The profile and distribution of these mutations. **(B)** The correlation analysis of these genes.

**FIGURE 2:**
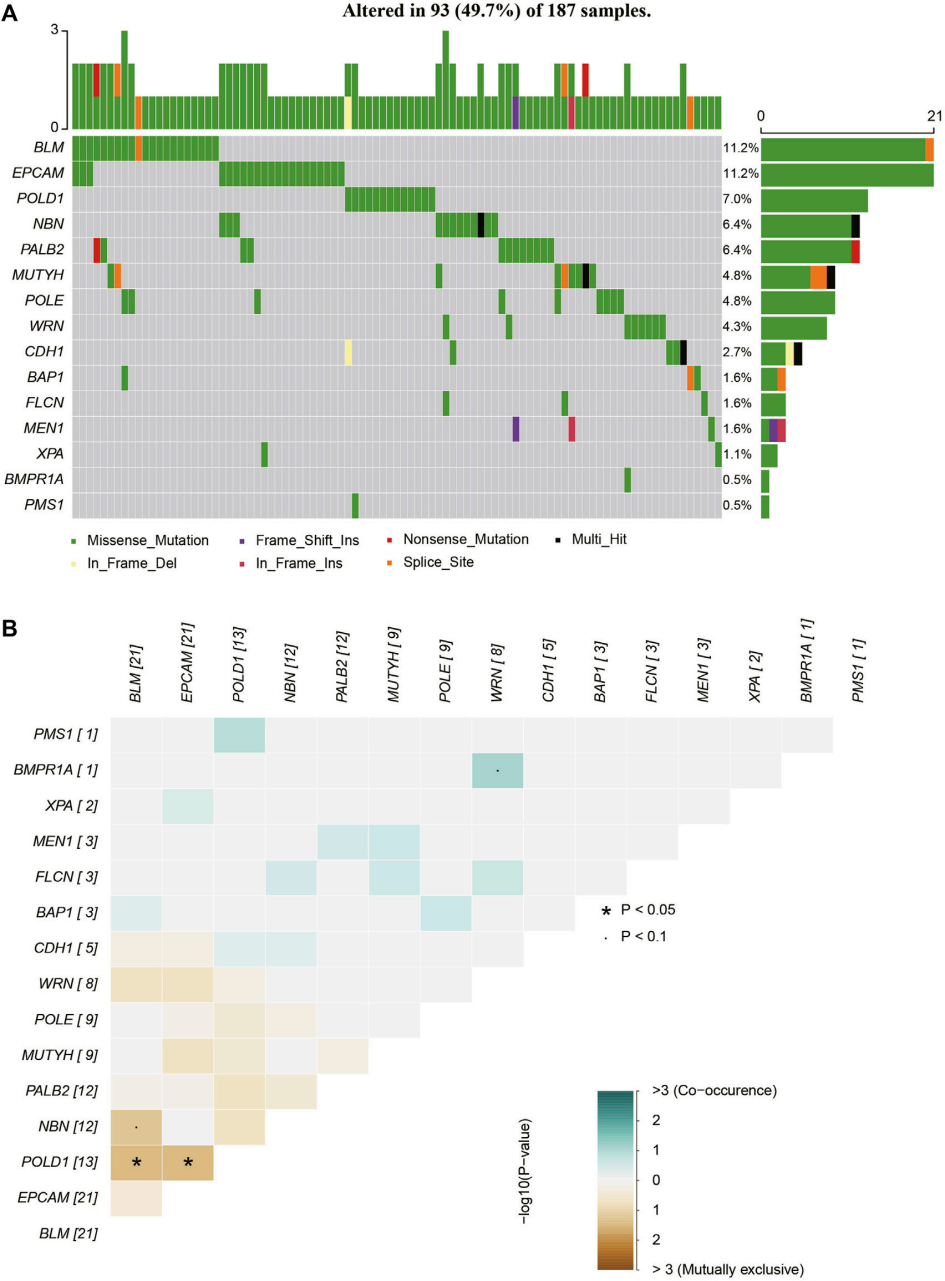
The mutations tested by 37-gene-panel in 187 cases. **(A)** The profile and distribution of these mutations. **(B)** The correlation analysis of these genes.

**FIGURE 3:**
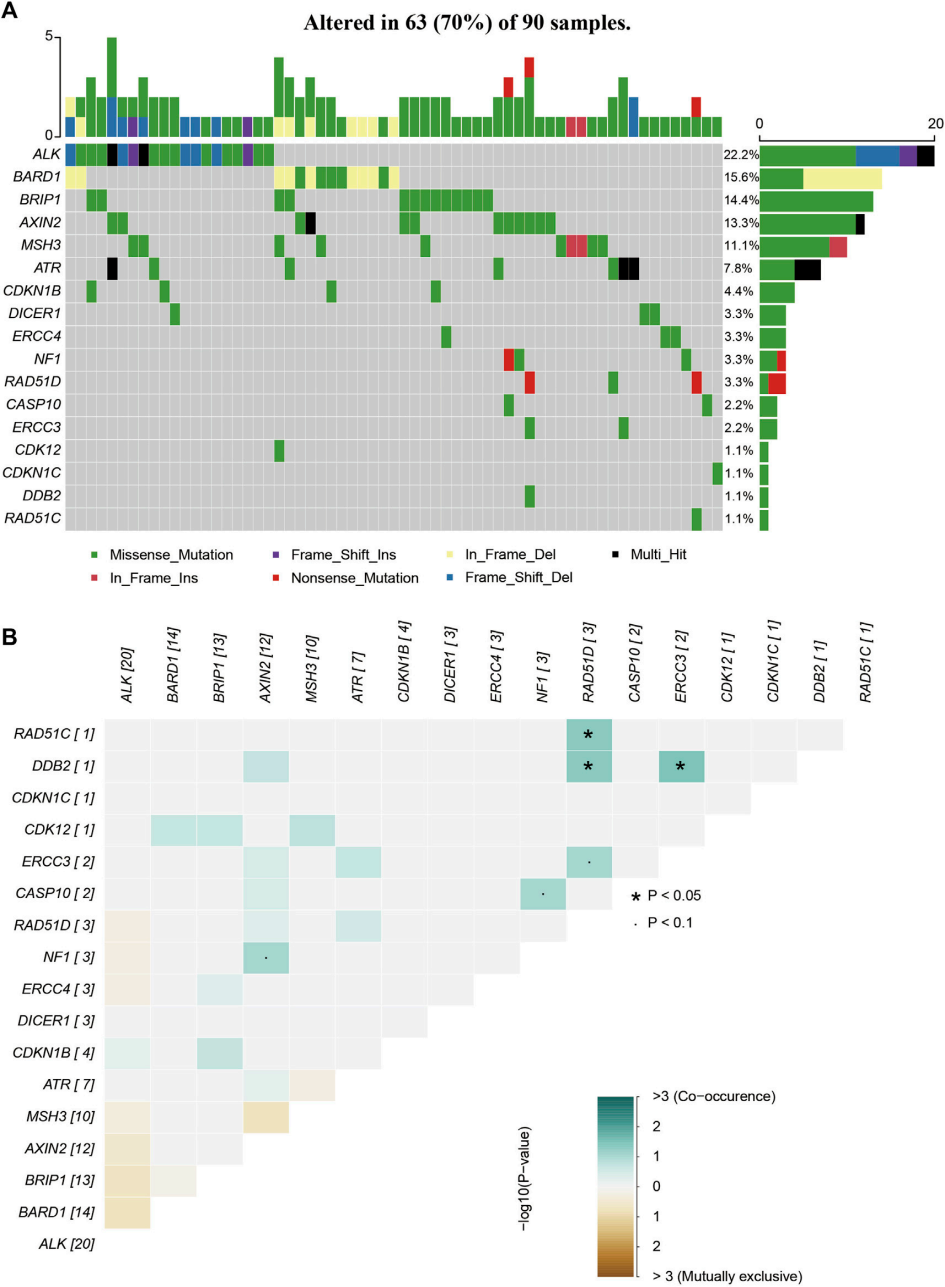
The mutations tested by 64-gene-panel in 90 cases. **(A)**The profile and distribution of these mutations. **(B)** The correlation analysis of these genes.

All rare variants identified within exonic and splicing regions were filtered based on an allele frequency of ≤0.05, as per the 1000 Genomes Project, ESP, and ExAC databases. A total of 421 rare variants were detected amongst the 253 patients with sporadic BC, consisting of 409 exonic and 12 splicing region variants. The exonic variants were classified into several types: 372 nonsynonymous single-nucleotide variants (SNVs), 7 stopgain, 11 frameshift deletions, 6 frameshift insertions, 5 nonframeshift deletions, 7 nonframeshift insertions, and 1 unidentified deletion. The frequency of rare variants within BC predisposition genes is outlined in Supplementary Table S2 and Figures 1–3. The most frequently mutated genes were *ALK* (20/90, 22.2%), *BARD1* (14/90, 15.6%), and *BRCA2* (38/253, 15.0%). We identified 14 unique *BRCA2* mutations in 38 cases (Figure 1A), with these mutations exhibiting a mutual exclusivity with *MLH1* mutations (Figure 1B, *p* < 0.1).

### ACMG level distribution of rare variants

**FIGURE 4:**
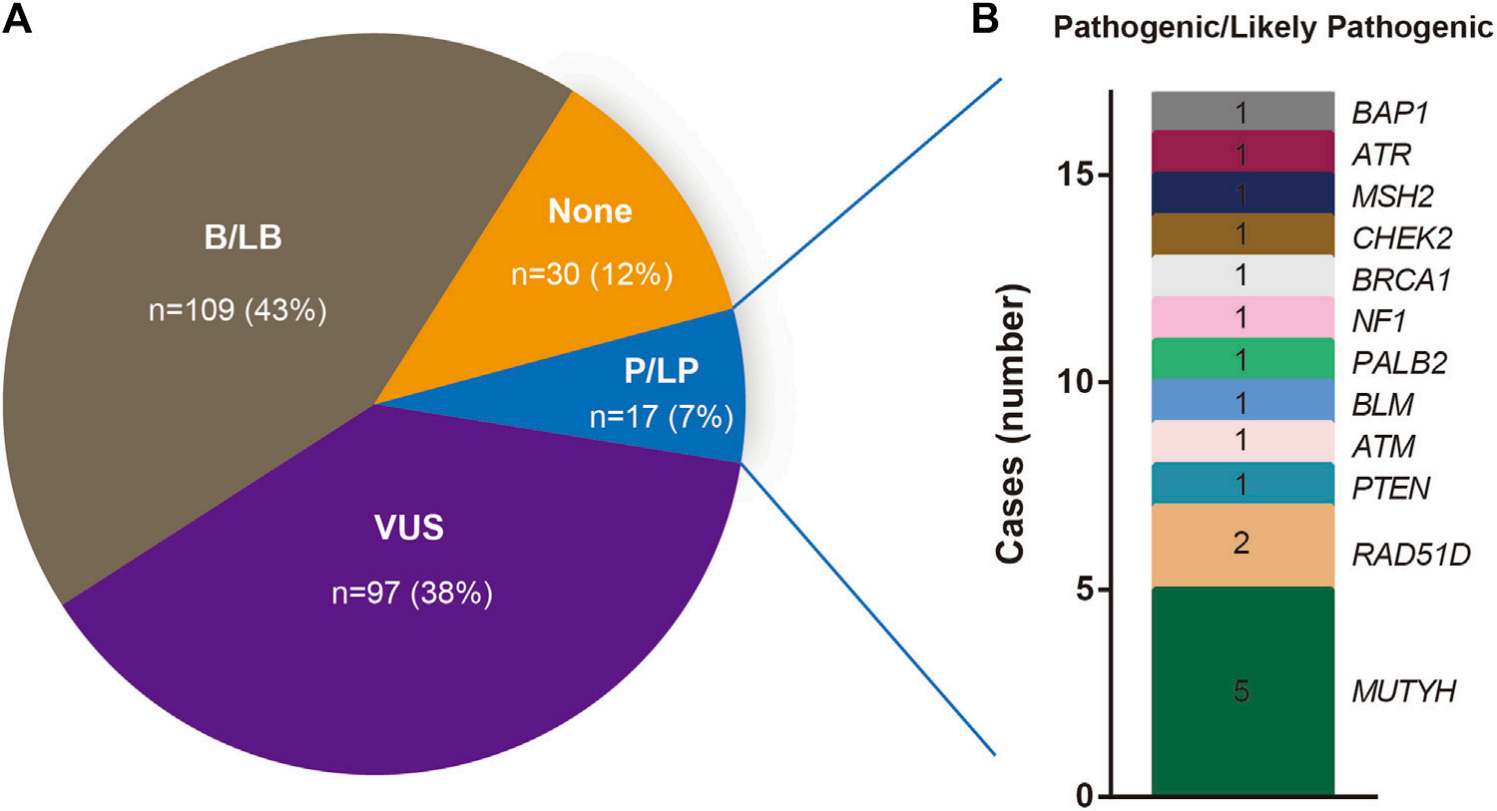
**(A)** Percentage of Beneficial/likely Beneficial (B/LB), Variants of uncertain significance (VUS), Pathogenic/Likely pathogenic (P/LP) and not recorded (none) mutations. **(B)** The number of the Genes that carry P/LP mutations.

Out of the 253 patients examined, 17 cases (17/253, 7%) carried pathogenic/likely pathogenic (P/LP) variants. A total of 97 patients (97/253, 38%) exhibited at least one variant of uncertain significance (VUS), without any P/LP variants, while 109 cases (109/253, 43%) harbored at least one benign/likely benign (B/LB) variant without any P/LP variants or VUS (Figure 4A). Notably, in 30 BC patients (30/253, 12%), no rare variants were detected.

### Distribution of pathogenic and likely pathogenic variants

The spectrum and details of the 17 identified pathogenic/likely pathogenic (P/LP) variants are depicted in Figure 4B and Table 2. To be specific, these P/LP variants include *NF1 c*.*C1381T*, *RAD51D c*.*C562T*, *PALB2 c*.*C2257T*, *MUTYH c*.*850-2A>G*, *BLM c*.*960-1G>A*, *PTEN c*.*211-2A>T*, *ATM c*.*2921+1G>T*, *BRCA1 c*.*5015delT*, *CHEK2 c*.*C409T*, *MSH2 c*.*C1566A*, *RAD51D c*.*C184T*, *ATR c*.*2320delA*, *BAP1 c*.*123-2A>C*, *MUTYH c*.*C689T*, *ATM c*.*1236-2A>T*. Of these, only one P/LP variant was found in a *BRCA* gene (specifically *BRCA1*), which was a frameshift deletion (*BRCA1 c*.*5015delT*) classified as a PV according to the American College of Medical Genetics and Genomics (ACMG). This variant was absent in the 1000 Genomes Project, ESP, and ExAC databases. The patient harboring this *BRCA1* PV had no family history of the disease and was initially diagnosed with triple-negative breast cancer (TNBC) at age 48, stage IIA. These findings further substantiate the potential pathogenicity of the *BRCA1 c*.*5015delT*, variant in sporadic BC cases. Additionally, of the 14 rare *BRCA2* variants, only one missense variant was classified as a variant of uncertain significance (VUS), with the remaining 13 variants categorized as benign or likely benign (B/LB).

**TABLE 2:** Pathogenic and likely pathogenic variants identified in multi-gene panel testing.

Gene	Alleles (ref/alt)	Mutation type	AA change	Sample count	1000 g	ExACALL	ExAC_EAS	ACMG Level
*NF1*	c.C1381T	stopgain	p.R461X	1	—	—	—	Pathogenic
*RAD51D*	c.C562T	stopgain	p.R188X	1	—	3.30E-05	0.0001	Pathogenic
*PALB2*	c.C2257T	stopgain	p.R753X	1	—	3.30E-05	—	Pathogenic
*MUTYH*	c.850-2A>G	splicing	—	2	0.00299521	0.001	0.0141	Pathogenic
*BLM*	c.960-1G>A	splicing	—	1	—	—	—	Pathogenic
*PTEN*	c.211-2A>T	splicing	—	1	—	—	—	Pathogenic
*ATM*	c.2921 + 1G>T	splicing	—	1	—	—	—	Pathogenic
*BRCA1*	c.5015delT	frameshift deletion	p.V1672fs	1	—	—	—	Pathogenic
*CHEK2*	c.C409T	stopgain	p.R137X	1	—	3.30E-05	0	Pathogenic
*MSH2*	c.C1566A	stopgain	p.Y522X	1	—	—	—	Pathogenic
*RAD51D*	c.C184T	stopgain	p.Q62X	1		8.33E-06	0.0001	Likely Pathogenic
*ATR*	c.2320delA	frameshift deletion	p.I774fs	1		0.0053	0.0039	Likely Pathogenic
*BAP1*	c.123-2A>C	splicing	NA	1				Likely Pathogenic
*MUTYH*	c.C689T	nonsynonymous SNV	p.A230V	3	0.00139776	0.0004	0.0047	Likely Pathogenic
*ATM*	c.1236-2A>T	splicing		1		2.76E-05	0	Uncertain significance

While *MUTYH* mutations are typically associated with colon cancer, increasing evidence suggests their pathogenic potential in BC (Chen et al., 2020; Felicio et al., 2021). In this study, two pathogenic/likely pathogenic (P/LP) *MUTYH* variants were identified in five BC patients (Table 2). The *MUTYH c*.*850-2A>G* variant, located in the splicing region, was found in two patients in our cohort. This variant exhibited allele frequencies of 0.003, 0.001, and 0.014 in the 1000 Genomes Project, ExAC_ALL, and ExAC_EAS databases, respectively. Classified as a PV by ACMG guidelines, it is commonly associated with MYH-associated polyposis. Among the two patients carrying this variant, one, a 49-year-old patient diagnosed with Luminal A breast cancer, had no family history of the disease. The other, a 63-year-old patient diagnosed with basal-like breast cancer, had a sister who was also a BC patient. The *MUTYH c*.*C689T* variant, found within the exonic region, was identified in three patients. This variant displayed allele frequencies of 0.0014, 0.0004, and 0.0047 in the 1000 Genomes Project, ExAC_ALL, and ExAC_EAS databases, respectively. While the ACMG guidelines list *MUTYH c*.*C689T* as a likely pathogenic variant (LPV), the ClinVar database classifies it as likely benign. Notably, none of the three patients harboring this variant had a family history of BC.

### Distribution of variants of uncertain significance in our cohort

A total of 97 distinct variants, annotated as variants of uncertain significance (VUS) and located across 39 different genes, were identified in 112 patients, as detailed in Supplementary Table S3. Of these patients, one individual harbored four VUS, nine patients carried three VUS, 29 patients exhibited two VUS, and the remaining 73 patients presented with a single VUS. Notably, the *PTEN c*.*C804A* variant displayed the highest frequency, appearing in 36 patients within our cohort. The allele frequencies of this variant were found to be 0.0009 and 0.0006 in the ExAC_ALL and ExAC_EAS databases, respectively. This significant contrast suggests a potentially critical role for this variant in the oncogenesis of sporadic BC. Furthermore, the *ALK c*.*2760_2766del* variant was found in six patients in our cohort. This variant’s allele frequencies in the ExAC_ALL and ExAC_EAS databases were 0.00008 and 0.0001, respectively. Intriguingly, all patients carrying the *ALK c*.*2760_2766del* mutation also harbored at least one other genetic mutation.

### Reclassification of ATM c.1236-2A>T variant as likely pathogenic based on ACMG criteria and HGMD data

The *ATM c*.*1236-2A>T* variant, currently classified as a variant of uncertain significance (VUS) by the American College of Medical Genetics and Genomics (ACMG), has been associated with cancer according to the high-confidence Human Gene Mutation Database (HGMD). Moreover, this variant is predicted to undergo nonsense-mediated decay, thus satisfying the PVS1 criterion for pathogenicity as per ACMG guidelines. Its low frequency in the ExAC_EAS database and its location within a highly conserved region further fulfill the PM2 and PP3 criteria, respectively. Accordingly, we propose a reclassification of *ATM c*.*1236-2A>T* as a LPV.

### Implications of rare mutations for their downstream protein structures

To identify significant rare mutations within the Chinese population, we conducted an in-depth investigation of all yet-to-be-reported variants without a reference number. Any variant that was classified as Benign or Tolerate by the predictive software SIFT, Polyphen2_HDIV, and Polyphen2_HVAR, failed to reach the threshold (ClinPred >0.95, and + CADD-based scores >25), located outside a functional domain, or were present in patients who already had P/LP variants were excluded from further analysis. The filtering process identified three rare variants for further investigation, specifically *ATM c*.*C8573T*, *CDKN1C c*.*C221T*, and *MSH3 c*.*A2723T*. To elucidate their biological implications, we performed *in silico* protein modeling analysis.

**FIGURE 5:**
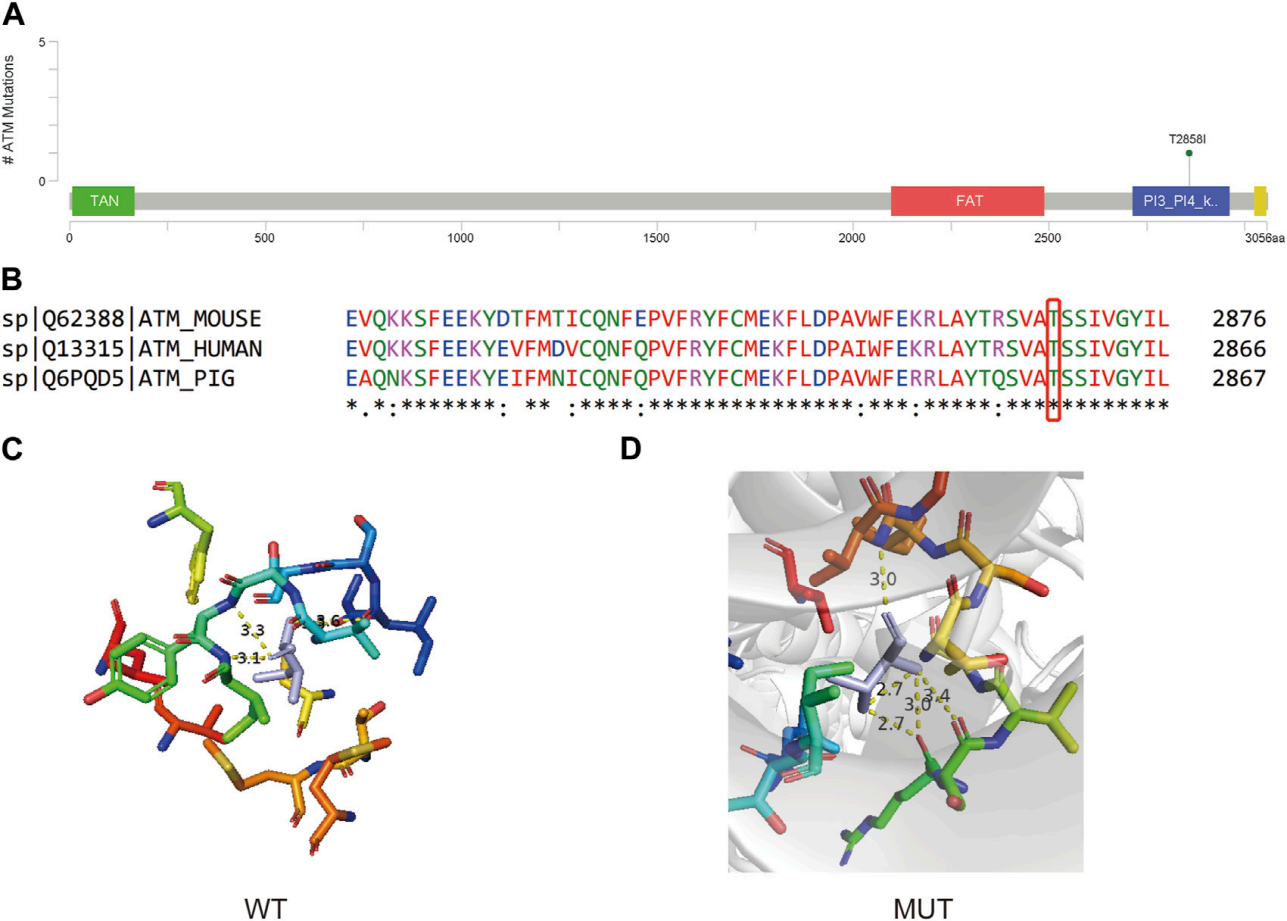
**(A)** Lollipop plot showing the location of *ATM c*.*C8573T* in ATM gene. **(B)** Protein conservation assessment of the amino acid affected by *ATM c*.*C8573T*, which is emphasize by a red box. **(C,D)** The *in silico* protein model of wildtype **(C)** and mutated type **(D)**.

The *ATM c*.*C8573T* variant resides in the Phosphatidylinositol 3- and 4-kinase domain, as illustrated in Figure 5A. This mutation engenders a nonsynonymous Threonine to Isoleucine substitution at the conserved position 2858, suggesting potential functional impact on the resultant protein (Figure 5B). 3D protein model prediction and energy change analysis indicate that the Threonine/Isoleucine substitution resulted in a reduced inter-atomic distance in the mutated domain (Figures 5C, D). This corresponds to a mutation Cutoff Scanning Matrix (mCSM) protein stability score of ΔΔG = −0.367 kcal/mol, indicating a potential decrease in stability.

**FIGURE 6:**
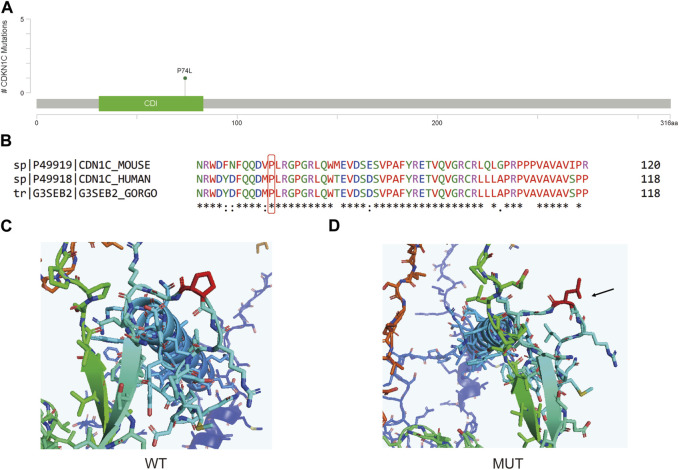
**(A)** Lollipop plot showing the location of *CDKN1C c*.*C221T* in CDKN1C gene. **(B)** Protein conservation assessment of the amino acid affected by *CDKN1C c*.*C221T*, which is emphasize by a red box. **(C,D)** The *in silico* protein model of wildtype **(C)** and mutated type **(D)**.

The *CDKN1C c*.*C221T* variant is situated in the Cyclin_dependent kinase inhibitor domain (Figure 6A), leading to a Proline to Leucine nonsynonymous mutation at a conserved position 74 (Figure 6B). Analysis of the predicted 3D protein model reveals a covalent linkage between the proline’s carbon atom and the protein structure, maintaining an intact aromatic ring in the wild-type protein (Figures 6C, D). However, this mutation disrupts the aromatic ring structure, resulting in a decrease in protein stability (ΔΔG = −0.618 kcal/mol).

**FIGURE 7:**
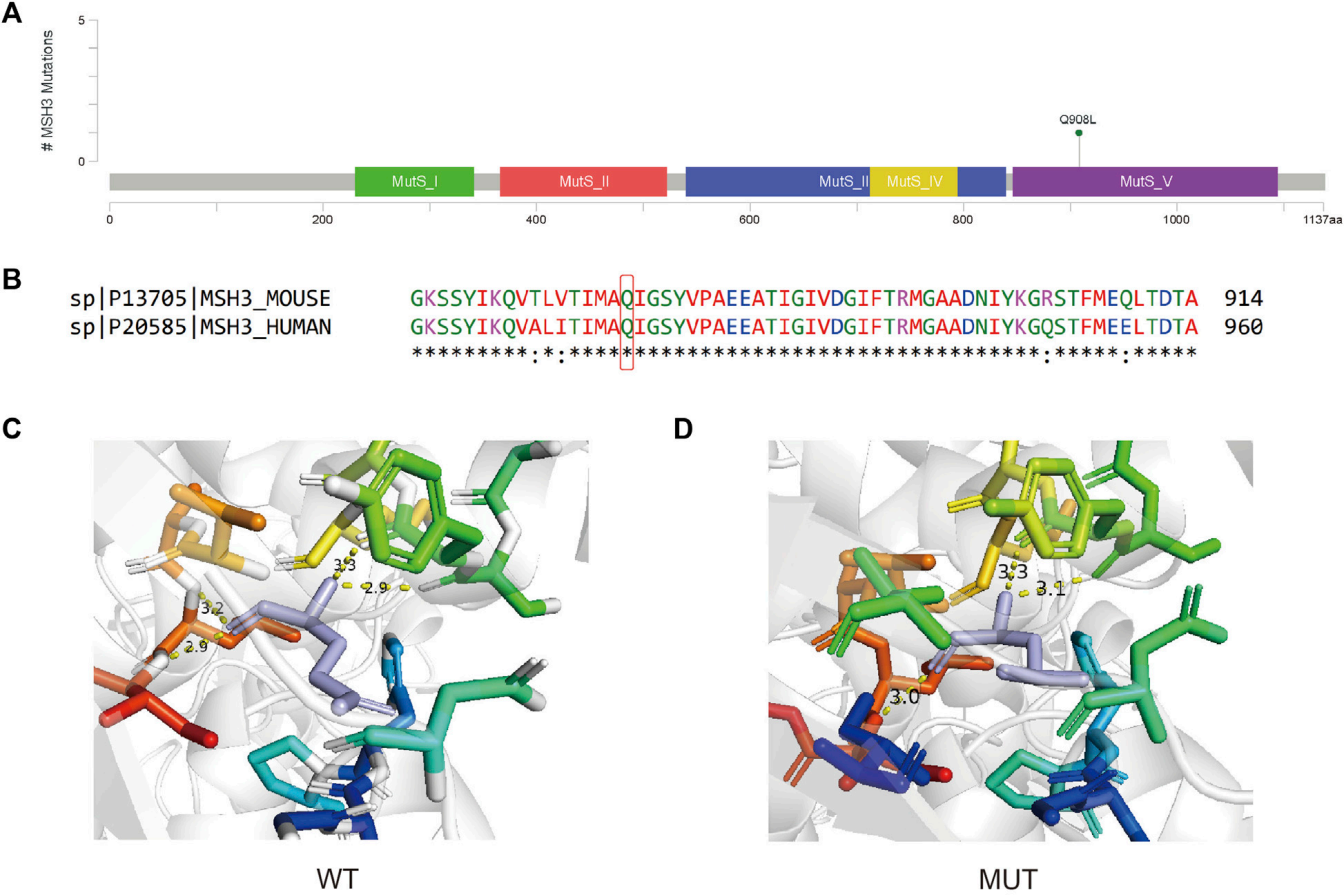
**(A)** Lollipop plot showing the location of *MSH3 c*.*A2723T* in MSH3 gene. **(B)** Protein conservation assessment of the amino acid affected by *MSH3 c*.*A2723T*, which is emphasize by a red box. **(C,D)** The *in silico* protein model of wildtype **(C)** and mutated type **(D)**.

The *MSH3 c*.*A2723T* variant is located within the MutS domain V (Figure 7A) and introduces a Glutamine to Leucine nonsynonymous mutation at protein position 908 (Figure 7B). This mutation, occurring at a conserved site, results in an unstable protein structure (ΔΔG = −0.324 kcal/mol) and alters the inter-atomic distances between several amino acids (Figures 7C, D).

## Discussion

In this study, we investigated the genetic landscape of BC in a cohort of 253 patients, identifying significant genetic variants that may contribute to disease susceptibility and progression. Our results, which highlight the heterogeneity and complexity of BC genetics, underscore the potential for personalized medicine in its management.

Most cases in our cohort (northern Chinese population) are sporadic BC, and the top 3 frequent mutated genes in were *MUTYH* (5/17, 22.2%), *BARD1* (14/90, 15.6%) and *BRCA2* (38/253, 15.0%), and the frequency of top 2 P/LP variants are *MUTYH* (5/187, 2.26%) and *RAD51D* (2/90, 2.22%). In Yi’s research, out of the 27 individuals studied, 9 (constituting 13.6% of the sample population) were identified as carriers of the *TP53* gene mutation, 5 (representing 7.6%) carried the *MSH6* mutation, and another 5 (equivalent to 7.6%) were found to carry the *BRCA1* mutation (Yi et al., 2019), while no P/LP variants were found. Another research on Chinese population focused on the prevalence and characteristics of *BRCA1/2* germline mutations, and *BRCA1/2* mutation shows low frequency (2%) in sporadic BC (Zhang et al., 2016). Through the analysis of our multi-omics TNBC cohort consisting of 325 individuals, Ma et al. mapped the range of germline variants in TNBC to assess their biological and clinical impacts, with the most common mutations identified in the genes *BRCA1* (7.4%), *RAD51D* (2.8%), and *BRCA2* (2.2%) (Ma et al., 2021). A research by Su et al. also reported the frequency of *RAD51D* is 1.3% in high-risk BC patients (Su et al., 2021). In Hongkong population, *RAD51D* is one of the most common mutation gene (0.8%) (Kwong et al., 2020). The frequency of *MUTYH* in Chen’s research is 0.7% (Chen et al., 2020), while in Jian’s research, it is 1.7% (Jian et al., 2017). By testing 30 cancer susceptible genes in 384 Chinese subjects with 2 high-risk factors, Lang et al. reported that both *MUTHY* and *RAD51D* have pathogenic/likely-pathogenic, with frequency of 2.9% and 0.5% (Lang et al., 2020). All these results suggest that genes that carry P/LP variants and their frequency are slightly different among Chinese population in different area, and may be affected by the high-risk factors of BC, indicating that the multi-gene test screening for BC patients are important any may found new target for the early screening or therapies.

*ATM* is a gene that involved in DNA double-strand break repair pathways, and usually the PVs in *ATM* are considered to be associated with a moderate risk of BC (Graffeo et al., 2022). The Phosphatidylinositol 3- and 4-kinase domain (also called *ATM* kinase domain) is a kinase domain that shares significantly homologous structure to that of *PI3K*, and activated ATM protein use this kinase activity to phosphorylate a series of downstream targets that are essential for DNA-damage repair (Phan and Rezaeian, 2021). The instability of the kinase domain caused by *ATM c*.*C8573T* (p.T2858I) may affect this domain and eventually the dysfunction of down-stream DNA-damage response. Although currently there is no record for this site in Clinvar, the nearby annotated mutation sites show the potential influence on the function of this protein. When only considering pathogenic, likely pathogenic, benign, and likely pathogenic, *ATM c*.*8564G>T* (p.S2855I) and *ATM c*.*8565T>A/G* (p.S2855R) are the closest mutation site near *ATM c*.*C8573T*, and are Likely pathogenic and Pathogenic/Likely pathogenic, respectively. A total of 14 Pathogenic/Likely pathogenic or Likely pathogenic and 1 Likely benign were found in this domain. A mutation on the same amino acid site *ATM*: *c*.*8572A>G* (p.T2858A) is annotated as uncertain significance. Hence, based on the current information, though the significance of *ATM c*.*C8573T* is not clearly identified, but based on the nearby mutation and the fact that it is the only mutation in that patient of our study, it may serve as a rare PV in at least Chinese population.

*CDKN1C*, is a gene that codes for the Cyclin‐dependent kinase inhibitor p57Kip2. This protein can obstruct the interaction domain on cyclins, preventing ATP binding and catalytic function, which in turn leads to the inhibition of the cyclin/CDK complex and slows down cell growth (Lai et al., 2021). As a gene associated with suppressing tumor growth, *CDKN1C* is linked to a variety of human cancers and Beckwith-Wiedemann Syndrome. Prior research has attempted to explore the relationship between *CDKN1C* methylation and BC (Zohny et al., 2017). However, how *CDKN1C* variants may affect BC oncogenesis is not well-studied. By searching the nearby variants of *CDKN1C c*.*C221T*, only one pathogenic, one likely pathogenic and one conflicting interpretations of pathogenicity annotation were found in this domain, and the conditions were all annotated to Beckwith-Wiedemann syndrome. The patient carrying this variant is a 57-year-old female with Luminal B breast cancer on stage IA. Though this variant is the only one she carries based on our testing panel, whether this variant somehow led to breast remain unclear.

*MSH3* is a crucial contributor to the mismatch repair (MMR) pathways, which is an essential biological process that exerts significant control over cell cycle regulation and apoptosis, thereby mitigating various forms of DNA damage (Brown et al., 2003). In the absence of appropriate repair mechanisms, these mismatches may increase spontaneous mutation rates, ultimately fostering microsatellite repeat instability in cells and promoting carcinogenesis. Some potentially functional variants of *MSH3* may influence the DNA repair capacity and thereby predispose individuals to a variety of cancers. For example, rs26279 (*MSH3 c*.*3133G>A*) has been frequently studied and implicated in carcinogenesis in recent years (Miao et al., 2015). However, the potential influence of *MSH3 c*.*A2723T* variant have not been recorded. Another mutation on the same site *MSH3 A2723C* (p.Gln908Pro) is related to Hereditary cancer-predisposing syndrome but is considered uncertain significance. The same situation can be found on all mutations that around this site. Hence, *MSH3 c*.*A2723T* may cause a potential effect on carcinogenesis of BC, but further studies are still needed.

While our study provides valuable insights into the mutation frequency among women with BC in northern Chinese population, it is not without limitations. First, our study focuses on a specific geographical location, which could limit the generalizability of our findings to other population groups. This is particularly important given the genetic heterogeneity of BC. Second, our sample size relatively small when considering the broad spectrum of BC patients globally, which might limit the statistical power of our results. Third, our study relies on genomic DNA extracted from peripheral blood and not from the tumor itself, which could potentially miss tumor-specific mutations and underestimate intra-tumor heterogeneity. Lastly, while we utilized stringent filtering criteria to identify relevant mutations, the potential for false-positive or false-negative findings remains, due to the inherent complexities of genomic analysis. Despite these limitations, our study lays crucial groundwork for further research on genetic predisposition to BC in this specific population.

## Data Availability

The datasets presented in this study can be found in online repositories. The names of the repository/repositories and accession number(s) can be found below: https://www.ncbi.nlm.nih.gov/, PRJNA998571.
